# Role of *N*-Acetylcysteine in the Treatment of Acute Nonacetaminophen, Nonalcoholic and Nonviral Hepatitis: A Meta-analysis

**DOI:** 10.1093/jcag/gwaa017

**Published:** 2020-07-15

**Authors:** Waleed Aljohani, Brian Pak Ho Chan, Mohammad Yaghoobi

**Affiliations:** 1 Department of Medicine, McMaster University, Hamilton, Ontario, Canada; 2 Division of Gastroenterology and Department of Health Research Methods, Evidence, and Impact (HEI), McMaster University, Hamilton, Ontario, Canada; 3 Gastrointestinal Health Technology Assessment Group, The Farncombe Family Digestive Health Research Institute, Hamilton, Ontario, Canada

**Keywords:** Acetylcysteine, Acute hepatitis, Cirrhosis, Cohort, Cohort analysis, Hepatitis, Liver failure, Liver transplant, Meta-analysis, NAC, *N*-acetylcysteine, Prospective study, Random, Randomized controlled trial (RCT)

## Abstract

**Introduction:**

*N*-acetylcysteine (NAC) has been extensively investigated for the use in acetaminophen and alcoholic hepatitis and is indicated in acetaminophen overdose. Studies assessing the effect of NAC on other forms of acute hepatitis in adult patients are limited and therefore here we aimed at evaluating the effect of NAC on survival in nonacetaminophen, nonalcoholic and nonviral hepatitis in adults.

**Methods:**

A comprehensive literature search up to September 2019 was completed for randomized controlled trials (RCTs) comparing NAC to placebo in the management of acute nonacetaminophen, nonalcoholic and nonviral hepatitis. Studies with insufficient data, non-RCT or nonprospective design, paediatric studies and studies with no comparator were excluded. Study selection, quality assessment and data extraction were independently performed by two co-authors. Primary outcome was survival. Secondary outcomes were an increase in infection rate. We used random model Mantel–Haenszel meta-analysis with Cochrane risk of bias to assess the quality of included studies. The recommendation was presented using the GRADE framework.

**Results:**

Seven out of 42 retrieved studies were included. Study population included patients with post-liver transplant, postsurgical, hypoxia-induced, ischemic and other nonalcoholic hepatitis. There was no difference in overall survival between NAC and placebo (odds ratio [OR] 0.95 [0.55 to 1.62]) in seven studies including 1033 patients. Furthermore, there was no difference in the rate of infection between NAC and placebo (OR 0.87 [0.43 to 1.79]). Random model analysis was used to adjust the effect of statistically significant heterogeneity in both analyses (*P* = 0.02). Lack of blinding in one study was found as a possible source of heterogeneity.

**Conclusions:**

NAC does not improve overall survival or the rate of infection in patients with acute nonacetaminophen, nonalcoholic and nonviral hepatitis as compared to placebo and should not be recommended in such setting which may even delay a transplant evaluation (level of evidence: 2a, GRADE of recommendation: B).

## Introduction

*N*-acetylcysteine (NAC) is an antioxidant that acts to counteract free radicals by increasing intracellular glutathione, especially in the liver (1–4). NAC optimizes cell protection, counterbalancing oxidative stress and inflammation (1–5). There are many clinical diseases have a benefit from NAC therapy, such as obstructive pulmonary disease, cystic fibrosis and systemic sclerosis (6–8). NAC has been extensively investigated for use in acetaminophen-induced hepatitis and is the first-line therapy for acetaminophen overdose (9). In alcoholic hepatitis, NAC infusion improved survival at 1 month when used as an adjuvant to prednisolone in a multicentre randomized controlled trial (RCT) but did not affect long-term mortality (10). Prednisolone and NAC were found to provide the best survival benefit at 28 days in a network meta-analysis (11,12). Despite this, the most recent *American College of Gastroenterology* guideline does not endorse the routine use of NAC given the paucity of studies (12,13). NAC showed no benefit in the treatment of acute viral hepatitis and with the advent of direct-acting antiviral medications, NAC is no longer a consideration in the treatment of viral hepatitis (13). Additional trials have compared the effect of NAC on varying etiologies of hepatitis, including ischemic, drug-induced, postsurgical and peri-transplant hepatitis. In this meta-analysis, we aimed at evaluating the effect of NAC on survival in nonacetaminophen, nonalcoholic and nonviral hepatitis in adults.

## MATERIALS AND METHODS

### Search Strategy

Electronic searches were conducted using OVID MEDLINE, EMBASE, Web of Science, Cochrane Library and Google Scholar. Recursive searches and cross-referencing were carried out by using a “similar articles” function. References of articles identified after initial search were manually reviewed. The search was not restricted to any specific language, abstract or country of origin. The following terms were used: acute hepatitis, liver failure, liver transplant, cirrhosis, hepatitis, acetylcysteine, n-acetylcysteine, cohort analysis, prospective study, cohort, random and randomized controlled trial (RCT).

### Study Selection

All RCTs and prospective cohort studies comparing NAC with placebo in the treatment of nonacetaminophen, nonalcoholic and nonviral hepatitis in adults up to September 2019 were included in this review. Literature search, study selection, data collection and quality assessment were conducted by two independents reviewers (W.A. and B.P.H.C.). A third reviewer (M.Y.) was involved if conflict occurred.

### Types of Outcome

Our primary outcome was survival, but we did not require survival to be the primary outcome of the included studies, as long as it was reported by investigators. Our secondary outcome was rate of infections which was defined as any reported infection during NAC administration.

### Inclusion/Exclusion Criteria

We include RCTs in adult patients with acute hepatitis, not due to acetaminophen, alcohol or viral hepatitis were included. Studies with a secondary treatment, nonextractable data, paediatric studies and duplicate publications were excluded. All studies in which NAC was combined with another therapy were excluded. There were no restrictions on dose, timing and route of administration of NAC.

### Publication Bias

**Figure 1.**
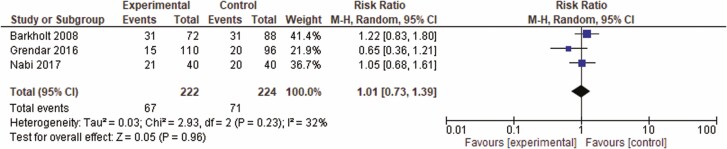
Risk of bias summary of included studies (green: low risk; yellow: unclear; red: high).

No restrictions were applied in terms of language, country of origin or quality of the studies. A funnel plot model was generated to explore the likelihood of publication bias (Figure 1).

### Reliability

In order to reduce selection bias, two independent reviewers (W.A., B.P.H.C.) performed the search, quality assessment and data extraction. A third reviewer (M.Y.) was involved when a consensus could not be achieved.

### Heterogeneity

Variation in the patient populations, different intervals of follow-up, different primary outcomes of each study and the quality of the studies were considered an a priori source of heterogeneity.

### Quality Assessment

**Figure 2.**
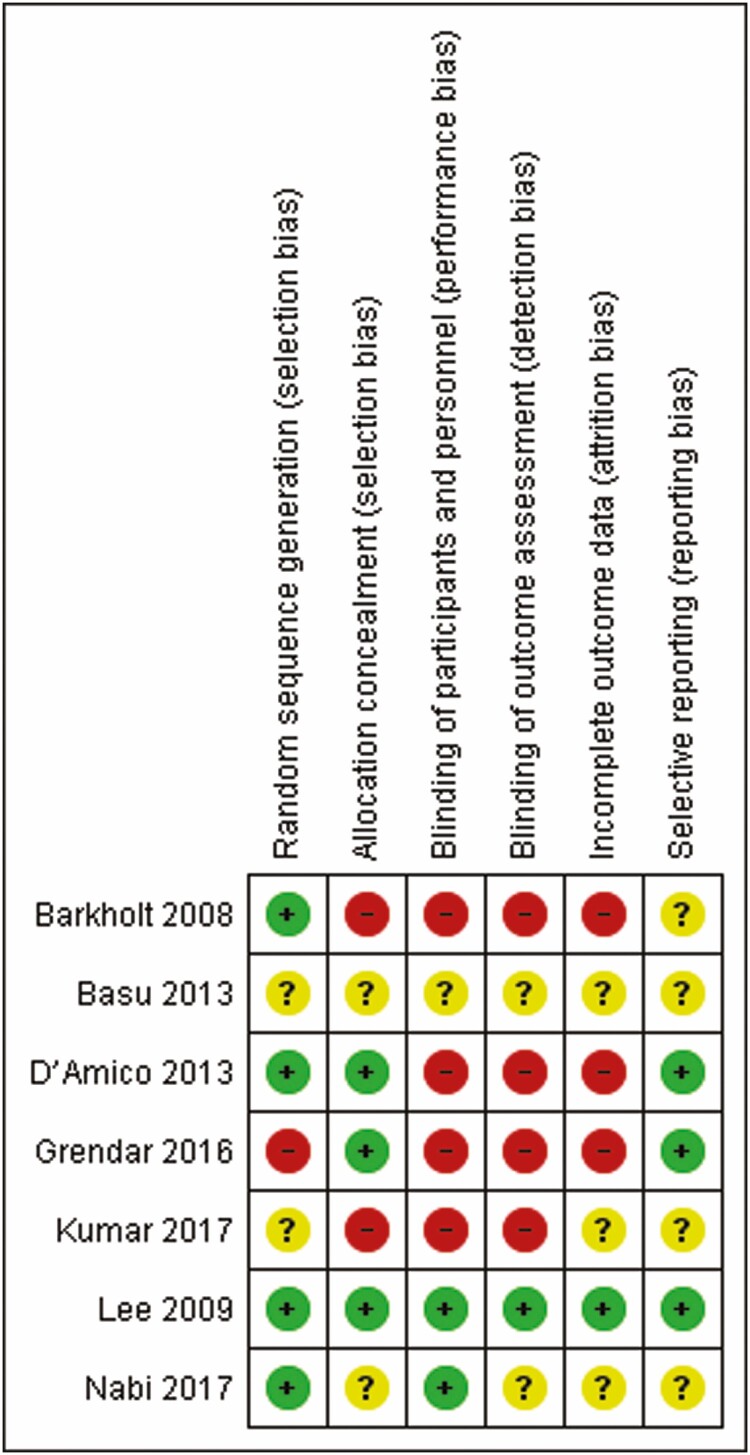
QUADAS-2 analysis, recommended by the Cochrane Collaboration for the assessment of risk of bias in included studies.

Quality of included studies was assessed using the Cochrane Tool for the Assessment of Risk of Bias for randomized trials. The Cochrane Tool for the Assessment of Risk of Bias addresses specific domains that are assessed as high risk of bias, low risk of bias or unclear including the following seven domains: sequence generation, allocation concealment, blinding of participants and personnel, blinding of outcome assessment, incomplete outcome data, selective outcome reports and other issues (14) (Figure 2).

### Subgroup Analysis

We performed subgroup analysis on trials conducted exclusively on liver failure patients versus those that included all ranges of hepatitis.

### Sensitivity Analysis

Jackknife analysis was performed by excluding each included study by turn to make sure to study has independently affected the result or the heterogeneity.

### Statistical Analysis

A meta-analysis of the pooled relative risk was performed using the Mantel–Haenszel method and Review Manager 5.3 (Version 5.3. Copenhagen: The Nordic Cochrane Centre, The Cochrane Collaboration, 2014). The random effect model was applied. A *P* value of 0.05 was applied as the criterion for statistical significance. The test of heterogeneity was considered significant if the *P* value was less than 0.10. All results were reported with 95% CI when applicable.

## RESULTS

**Figure 3.**
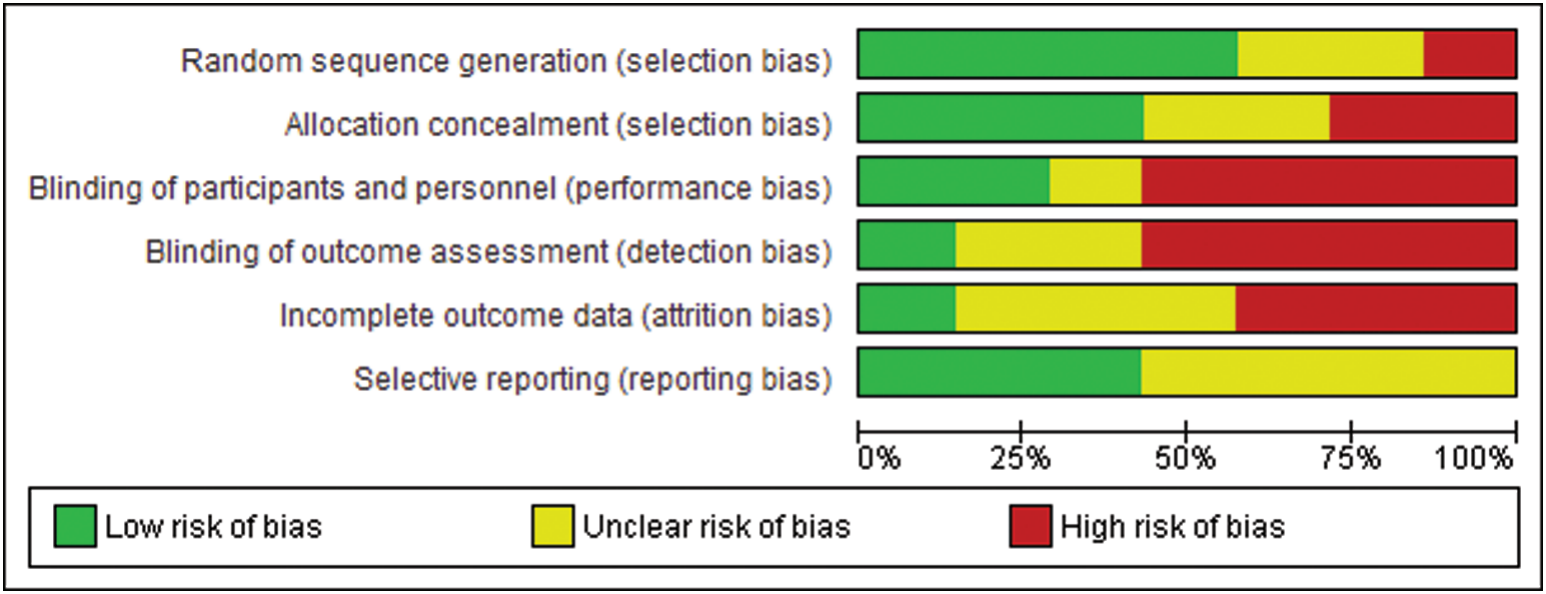
PRISMA (preferred reporting items for systematic reviews and meta-analyses) flow diagram of study identification, inclusion and reasons for exclusion.

**Figure 4.**
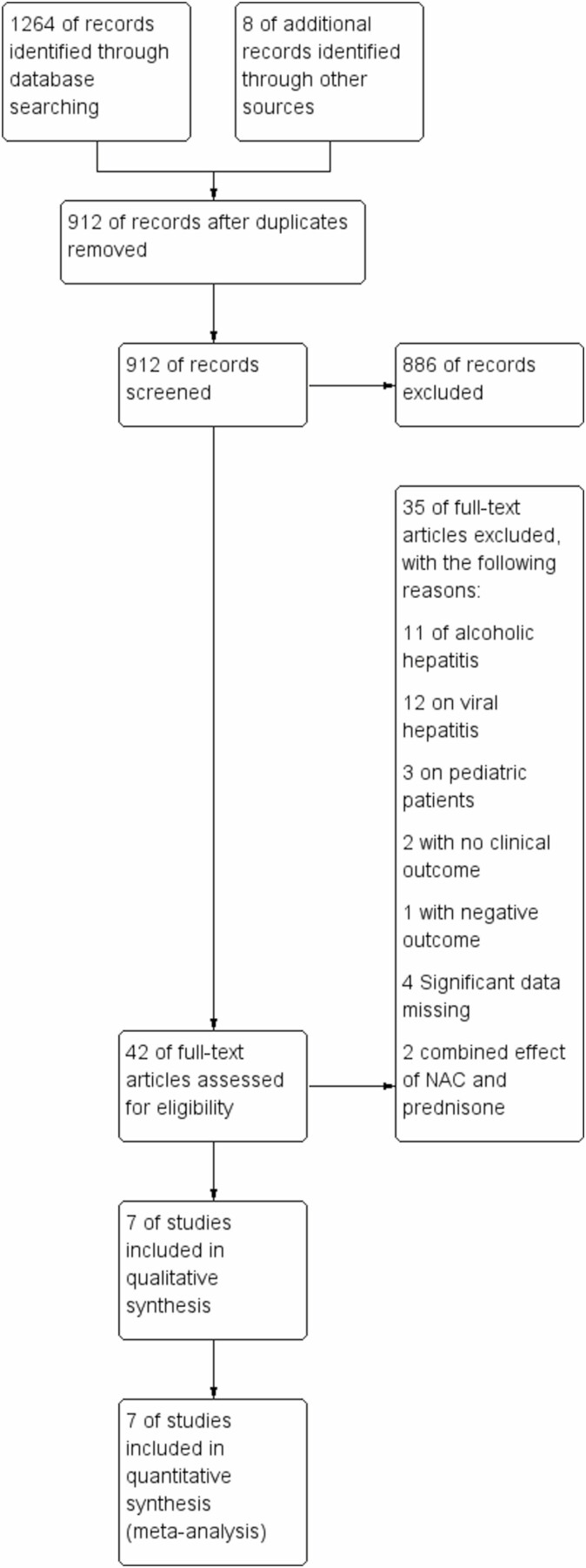
Funnel plot of included studies for the primary outcome of survival. SE = standard error; OR = odds ratio.

**Figure 5.**
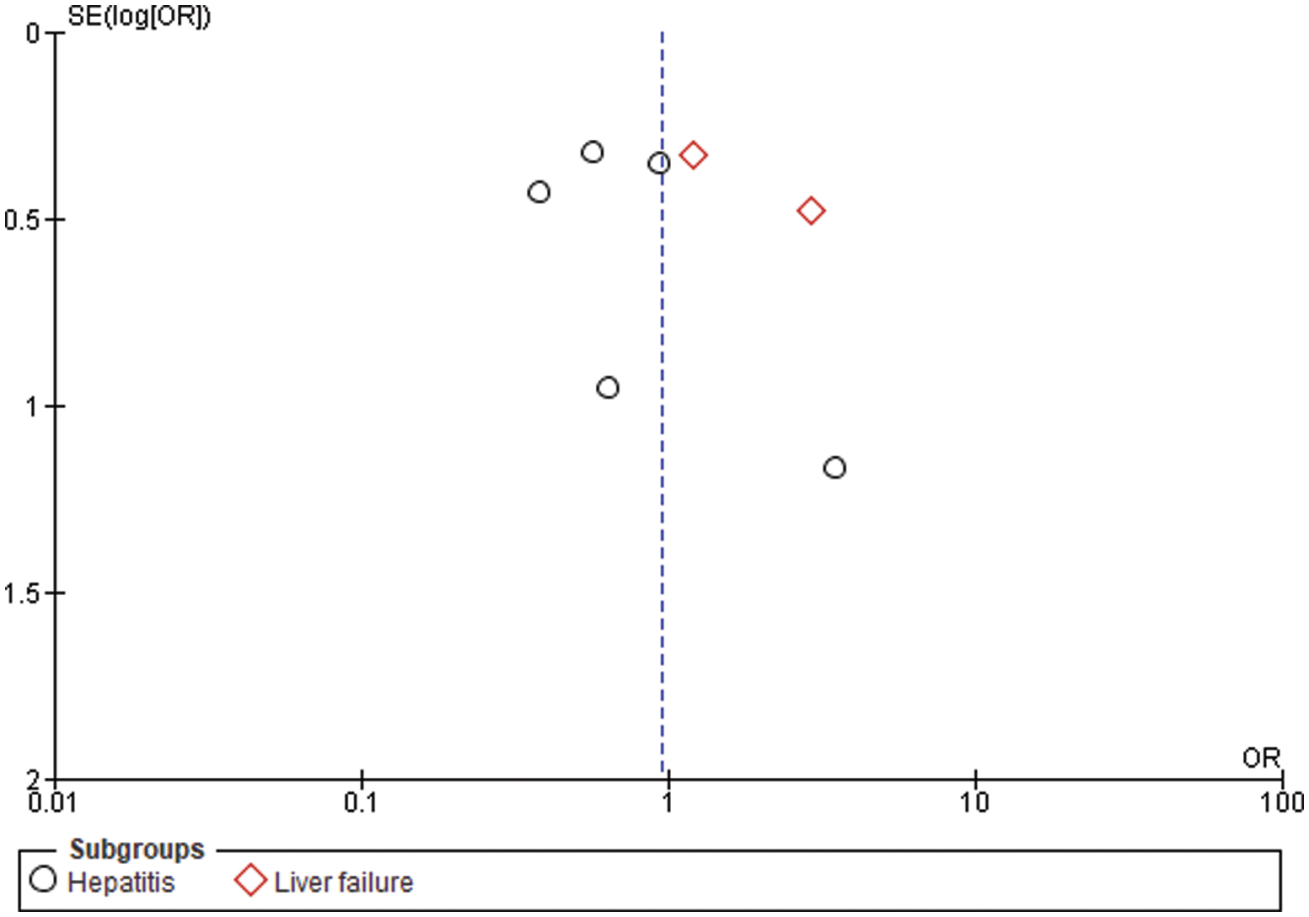
Forrest plot of included studies for overall survival. CI = confidence interval.

**Figure 6.**
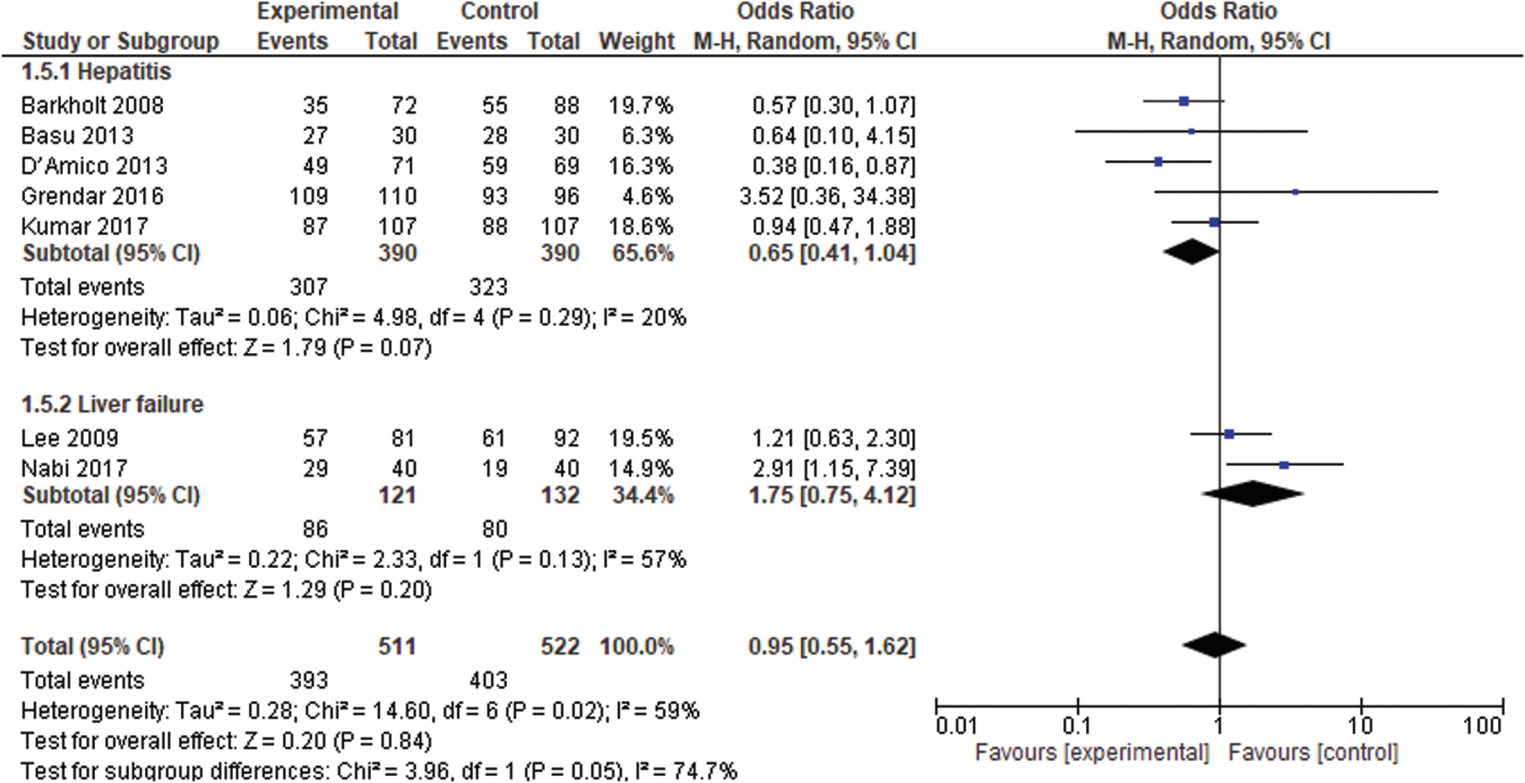
Forrest plot of included studies for rate of infection. CI = confidence interval.

A total of four abstracts and 44 full texts out of 1264 potential studies were reviewed, among which, seven studies including 1033 patients were included in the final analysis. The included studies included patients with post-liver transplant, postsurgical, hypoxia-induced, ischemic and nonalcoholic acute hepatitis. Additional details and study flow are depicted in a PRISMA flow diagram (Figure 3). Two of the included studies were only presented in abstract form. Table 1 shows the characteristics of the included studies. No visual asymmetry was observed in the Funnel plot. Figure 4 depicts the Funnel plot of the study. Among the seven studies presenting survival rate in a total of 1033 patients, NAC was not statistically different from placebo in improving survival (odds ratio [OR] 0.95 [0.55 to 1.62]; *P* = 0.84). The average survival rate for 1033 individuals with nonacetaminophen, nonviral and nonalcoholic hepatitis was 76.9% (393/511) as compared to 77.2% (403/522) in the placebo group. There was significant heterogeneity associated with this analysis (*P* < 0.02, *I*^2^ = 59) (Figure 5). There was no significant change in survival related to the severity of liver condition. Subgroup analysis in hepatitis patients showed that the survival rate was 99.3% (307/309) for those who received NAC as compared to 82.8% (323/390) in the placebo (*P* < 0.29, *I*^2^ = 20). In the hepatic failure subgroup, the survival rate was 71.07% (86/121) in the treated patients as compared to 60.6% (80/132) in the control group (*P* < 0.13, *I*^2^ = 57) (Figure 5). NAC was not significantly different from placebo in decreasing the rate of infection (OR 1.01 [0.73, 1.39], *P* = 0.71) in three studies including 464 patients. Pooled rate of infection was 30.1% in the NAC group and 31.6% in the control group. This analysis was also associated with significant heterogeneity (*P* < 0.23, *I*^2^ = 32%) (Figure 6).

**Table 1.** Characteristics of included studies

Study	Year	Design	Blinding	Abstract only	Population	*N* (NAC)	*N* (Placebo)
Barkholt et al. (15)	2008	RCT	No	No	Post-ASCT	72	88
Lee et al. (16)	2009	RCT	Double	No	Nonacetaminophen liver failure	81	92
Basu et al. (17)	2013	RCT	Unspecified	Yes	HILI	30	30
D’Amico et al. (18)	2013	RCT	Single	No	LT—donor	69	71
Nabi et al. (19)	2017	RCT	No	No	Nonacetaminophen liver failure	40	40
Grendar et al. (20)	2016	RCT	No	No	Hepatic resection	96	110
Kumar et al. (21)	2017	RCT	No	Yes	IH post-variceal bleed	107	107

ASCT, allogeneic stem cell transplant; HILI, hypoxia-induced liver injury; IH, ischemic hepatitis; LT, liver transplant; *N*, number; NAC, *N*-acetylcysteine; RCT, randomized controlled trial.

### Sensitivity and Subgroup Analyses

Nabi et al. (19) showed the highest heterogeneity effect and heterogeneity decreased (*I*^2^ = 34%) by removing this study in the Jackknife analysis. Furthermore, excluding the study by Grendar et al. decreased the heterogeneity from *I*^2^ = 20% to *I*^2^ = 0%. Lack of blinding and early termination due to higher rate of the development of delirium in case group as compared to the placebo (9.8% and 2.7%, *P* < 0.05) could explain its contribution to heterogeneity. We conducted subgroup analysis based on the presence or absence of liver failure that had no significant effect on heterogeneity.

## Discussion

To our knowledge, this is the first comprehensive systematic review and meta-analysis specifically conducted to address the value of using NAC in patients with nonacetaminophen, nonalcohol and nonviral causes of acute hepatitis. NAC did not improve survival, infectious complications or length of hospital stay in the management of hepatitis not related to acetaminophen, alcohol or viral infection to affect the survival rate. NAC is currently only approved for use in acetaminophen overdose and as a mucolytic (22). Given the proposed mechanisms of action, it has been widely studied (1–4). Our study excluded acetaminophen toxicity, as NAC is already first-line therapy in this condition. Alcoholic hepatitis was excluded as this has been extensively reviewed previously (23,24). Our study looked at post-liver transplant, postsurgical, hypoxia-induced, ischemic and nonalcoholic hepatitis, where there is a paucity of data on the role of NAC. Outside of supportive care as a bridge to transplant, there is a lack of treatment options in our patient population, which was the impetus of our meta-analysis. In the ACG guideline for the management of idiosyncratic drug-induced liver injury (16,25), no definitive therapies were available; however, NAC was listed as a consideration given its good safety profile. While our results do not support the use of NAC, there is a lack of published data and larger studies are required. NAC has been studied in other settings not included in this meta-analysis. In a prospective, multicentre observational study, NAC infusion was administered in acute liver failure patients without clinical or historical evidence of acetaminophen (26). Use of a NAC infusion reduced nonacetaminophen-induced acute liver failure mortality and need for transplantation. In addition, NAC decreased encephalopathy, hospital stay, ICU admission and failure of other organs. An RCT by Pamuk et al., comparing NAC versus controlled group who did not receive any treatment for nonalcoholic steatohepatitis showed an improvement in transaminases over 4 weeks (24).

The main limitation of our meta-analysis was heterogeneity. Significant statistical heterogeneity was present in all analyses and was likely due to clinical heterogeneity such as different aetiologies of hepatitis. We used a random effects model and sensitivity analysis to adjust for heterogeneity. Exclusion of three studies with a high risk of bias significantly reduced heterogeneity. This demonstrated that trial quality was the most likely reason for high heterogeneity.

Clinical heterogeneity is inevitable in meta-analysis especially in those topics where included studies provided controversial results causing statistical heterogeneity. Interestingly, controversial topics may benefit the most from a meta-analysis that could eventually provide more definite conclusion. In an optimal situation, one would like to include studies, which are as similar as possible in methodology, however, due to the nature of meta-analysis being dependent on available studies; this was not possible in our study. We attempted a subgroup analysis but we were not able to explain heterogeneity based on severity of disease in the included patients. Our Jackknife analysis showed that removing the study by Nabi et al. had the highest improvement in heterogeneity (19). This could have been related to the following factors: (i) Acetaminophen-induced hepatitis was excluded based on history with no biochemical confirmation. (ii) The duration of follow-up used to establish the survival rate was based on hospital admission until discharge from the hospital with no further follow-up. Also higher risk of bias might have been a source for heterogeneity since it decreased by excluding a nonblinded underpowered study (20). To minimize the effect of heterogeneity we used random model meta-analysis and the results of the meta-analysis remained robust despite high heterogeneity. We optimally would need individual patient data to further scrutinize the effect of heterogeneity and to study the benefit of NAC especially if the etiology could be identified clearly.

In conclusion, our meta-analysis showed no benefit in the use of NAC in the treatment of nonacetaminophen, nonviral, nonalcoholic hepatitis unless a high-quality study proves otherwise in the future. The studies included were of high heterogeneity with moderate risk of bias. Administrating NAC should not be a substitute for early referral to a transplant centre for any patient demonstrating evidence of hepatic failure.

## Funding

Dr. Yaghoobi’s research is partly supported by an Internal Career Award by the Department of Medicine at McMaster University.
